# Deriving expert-driven seismic and wind fragility functions for non-engineered residential typologies in Batanes, Philippines

**DOI:** 10.1038/s41598-023-49398-2

**Published:** 2023-12-12

**Authors:** Arvin Hadlos, Aaron Opdyke, S. Ali Hadigheh

**Affiliations:** School of Civil Engineering, The University of Sydney, Camperdown, NSW 2006 Australia

**Keywords:** Civil engineering, Natural hazards

## Abstract

Natural hazards inflict significant damage to dwellings in the Philippines where housing is often the most valued asset of households. Residential fragility functions estimate structural damage to mitigate risk but these are challenging to derive when empirical and analytical data are lacking, as is common in rural areas. Too often, conventional fragility estimates overlook the characteristics of informally built or non-engineered dwellings common in rural areas. We used a heuristic alternative of deriving fragility functions relying on experts’ judgements to understand the housing performance of non-engineered residential typologies in the Province of Batanes in the Philippines. Drawing on field surveys in the Municipality of Itbayat, we identified and defined seven prominent typologies. Based on the Applied Technology Council’s expert-driven method of deriving fragility functions, 18 experts estimated the damage states of these typologies against the impacts of earthquakes and typhoons which are the two most prominent hazards in the region. Our findings provide first-generation fragility functions for Batanes as a step towards more localised risk assessment in the Philippines. More broadly, these functions can be used for typologies identified beyond Batanes where similar structural characteristics are prevalent.

## Introduction

Building back safer after disasters has been a consistent mantra for post-disaster reconstruction efforts under the Sendai Framework for Disaster Risk Reduction^1^. Dwellings in low- and middle-income countries are the most valued assets of households but these also tend to be the most disaster-affected^2^. To safeguard these assets, it is imperative to understand their structural performance against the impacts of hazards. The derivation of fragility functions captures structural performance of a building when it is subjected to an environmental excitation^3–6^. Fragility functions, often depicted graphically as a series of fragility curves, appraise risk by quantifying the relationship between a hazard intensity (e.g., seismic intensity or wind speed) and the probability of a component or structure reaching or exceeding a certain damage state^3–6^.

The availability of fragility functions can support modelling natural hazard impacts and more informed targeting of structural safety programs. For example, deriving these functions can reveal vulnerable structural typologies in a building stock leading to risk prioritisation interventions. Hence, risk and loss estimation methods are usually anchored on deriving these functions, such as those developed by the United States Federal Emergency Management Agency (FEMA)^4,7–9^. However, in the context of resource-constraint communities, the limited data on past disaster damage and unregulated construction practices inhibit the derivation of fragility functions. While these functions are a foundational tool to understand structural safety and are useful for disaster preparedness, they are too often lacking in hazard-prone regions where they are critically important^10^.

In rural areas in low- and middle-income countries, dwellings built informally by residents are common. These are either patterned from longstanding vernacular practices, borne out of the immediate need to have shelter (e.g., urgent reconstruction due to disaster impacts), or as a compromise to rising construction costs. In these cases, the construction of dwellings is usually non-engineered, with the absence or limited oversight of qualified building professionals^11^. As a result, housing typologies often exhibit a high variance of characteristics compared to conventional building typologies designed and constructed based on prevailing building codes and standards. While these non-engineered structures are not prejudiced to be deficient in structural safety and may, in fact, reveal sound locally developed building practices^12^, their structural performance is less documented and certain. Furthermore, the exposure of dwellings to multiple natural hazards hinders our understanding of their performance when assessed against competing impacts on structures. Building-level disaster risk reduction (DRR) measures have shown how multi-hazard trade-offs and asynergies complicate building more resilient dwellings. That is, where construction practices may reduce the impact of risk to one hazard, such can exacerbate the risk to another hazard^13,14^.

Depending on the available sources of damage data, fragility functions can be derived in various ways^5,15^. When documented post-disaster damage data is available, empirical methods can be used. If resources allow for the simulation or modelling of structural typologies, analytical methods can be employed. When both resources are lacking, a heuristic alternative is to solicit experts’ opinions to derive the functions. A fourth approach is a hybrid of at least two methods. Further discussion about these four methods, including an overview of past studies using specific approaches, can be found in Maio and Tsionis et al.^16^ and Rossetto et al.^17^. Previous attention to deriving these functions has primarily focused on a structure’s performance to a single hazard. Recently, there has been a growing emphasis in academic, policy, and practice environments to incorporate multi-hazard approaches to realise effective DRR strategies^14,18,19^.

Hazards impact a structure differently thereby requiring a combination of strategies and approaches to reduce or mitigate risk^13,14^. Deriving multi-hazard fragility functions can therefore offer insights on how to optimise trade-offs in construction decisions. The scarcity of data and technical resources are, however, roadblocks in rural contexts in low- and middle-income countries. The expert-driven approach is often the only possible method in the absence of empirical and analytical data^16,20–22^. Elicitation of experts’ opinions has been used in various disciplines to produce the fragility functions of components of interests, extending from structural engineering^20,23–25^, hydrology^10,22^, and civil engineering^26^. The ATC-58 project of the Applied Technology Council (ATC) aimed to develop next-generation seismic design assessment standards, and the expert-driven method is adopted among other approaches to derive fragility functions as formalised through a proposed method of solicitation and aggregation of experts’ opinions^27,28^. The main strength of this approach is that experts can include in their assessment all factors affecting the response of a structure against hazards, unlike empirical and analytical methods where these are limited to the quantity and quality of available data^15^. The major drawback of expert-driven methods is the subjectivity associated with experts’ experience and trust in their judgement^15^. However, when dealing with non-engineered residential typologies, experts’ experience is beneficial to capture the varied structural performance arising from the inherent variability of the design and construction of these building classes.

The objective of this study was to derive fragility functions for non-engineered residential typologies in the Province of Batanes—the storm-battered northernmost part of the Philippines. We drew on field surveys in the Municipality of Itbayat to identify relevant typologies for the Province of Batanes. The remote island municipality of Itbayat was home to vernacular stone-and-lime houses, like elsewhere in Batanes, built out of tradition to withstand typhoon impacts. Unfortunately, these dwellings were extensively damaged following the series of destructive earthquakes (magnitudes (M_w_) 5.4, 5.9, 5.8) in 2019^29,30^. These disasters redefined the construction practices in the area with a departure from the stone-and-lime character of the building stock. We surveyed the existing building stock three years later and identified the most prominent typologies that households chose to build to replace these vernacular dwellings. Based on the expert-driven approach of deriving fragility functions developed in ATC-58, a pool of experts estimated the seismic and wind performance of both the vernacular and replacement typologies.

The rest of the paper is structured as follows. The Methods section characterises the research procedures and protocols undertaken for this study. Next, under the Results section, we present the identified housing typologies and the derived fragility functions. The Discussion section then provides insights gleaned in using the expert-driven approach in deriving fragility functions. Lastly, in the Conclusion section, we summarise the theoretical and practical implications of this study.

## Methods

Below, we outline how we surveyed the housing typologies, followed by the process of soliciting and aggregating experts’ estimates. We then present how we derived the fragility functions. Lastly, we provide information on the research ethics protocols and permits obtained for this study.

### Identification of housing typologies

Identifying a building typology is the first step in building-level risk estimation as this serves as the object of analysis for fragility functions. A typology, usually labelled with a taxonomy (string), characterises a building class from attributes posing vulnerability to the impacts of natural hazards^4,31^. For example, for earthquake risk, structures are classified according to the (i) type of lateral load-resisting system, (ii) material of lateral load-resisting system, (iii) building height, and (iv) seismic code level (or the period of construction of a structure vis-à-vis the enforcement of seismic regulations). Such attributes are the core parameters used in risk analysis by many organisations, such as FEMA for HAZUS^7^ and the Global Earthquake Model (GEM)^31^. For typhoon risk, the same set of attributes are relevant, but with wind-induced damage concentrated on walls and roofs^32,33^, building envelope materials and roof profile are usually considered in conjunction^34^. In this study, we used all these attributes—except for the seismic code level—to inform the development of a rapid visual survey to assess the attributes of a building stock. The seismic code level was omitted because the housing typologies surveyed were non-engineered, with formal seismic regulations having limited applicability to these types of structures.

**Figure 1:**
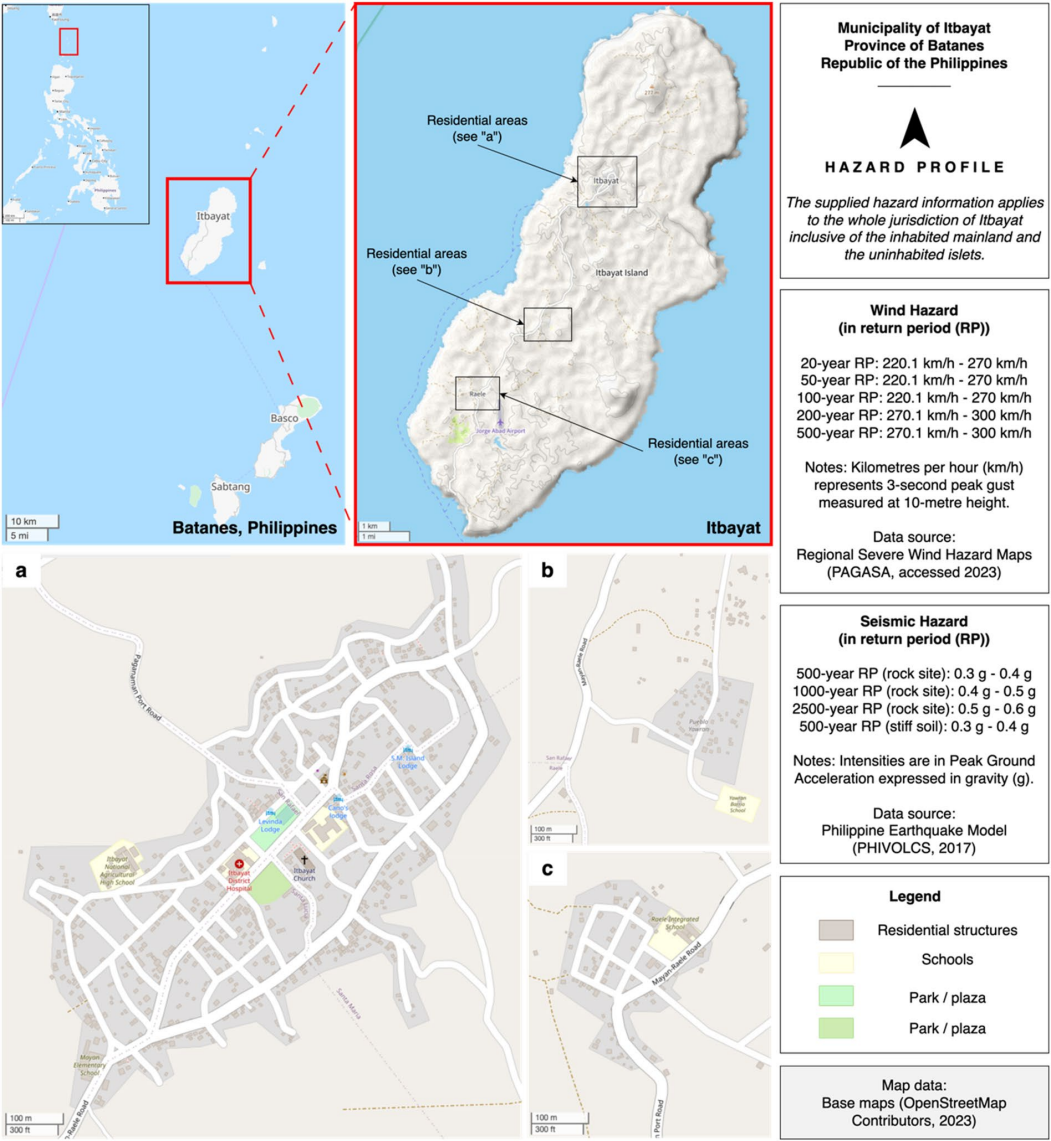
Map of Itbayat, Batanes in the Philippines.

The rapid visual surveys were conducted in January 2023 using the five building attributes as the parameters of the assessment. For lateral load-resisting systems, we referred to the expanded classifications and definitions of the GEM taxonomy^35^ since their database accounts for the structural characteristics of non-engineered construction^31^. The field study aimed to understand the housing reconstruction strategies of households after the 2019 earthquakes in the Municipality of Itbayat, located in the Province of Batanes in the Philippines (see Fig. 1). Hence, we focused the surveys on the emergent (or replacement) typologies constructed by households who were living in vernacular stone-and-lime houses before the earthquakes but were forced to rebuild due to the extensive damage to this typology. This pre-earthquake typology was also surveyed. Out of the 153 households identified from the joint report of the Municipal Disaster Risk Reduction Office (MDRRMO) and the Municipal Planning and Development Office (MPDO) in Itbayat, we were able to survey 101 structures. The remaining houses were not surveyed due to the unavailability of households despite multiple attempts at the time of the survey (n = 35) or where households did not reconstruct their houses and have either migrated to a different municipality or living with relatives (n = 17). In surveying the attributes of the stone-and-lime housing typology, we relied on households’ recollection about the features of their past dwelling. We also triangulated these features via archival research (e.g., photo documentation from the National Commission on Indigenous Peoples (NCIP)), desk research (e.g., publications^36–40^), and field visits to a few standing archetypes in the municipality. For the emergent housing typologies after the earthquakes, these were surveyed as all the necessary information was observable onsite.

### Solicitation of experts’ opinion

After identifying the predominant housing typologies, we selected a pool of experts to estimate the damage states of these typologies. In qualifying “expertise”, we recognised both the inputs of those who are locally based (“local experts”) and those who are not but with relevant insights on the housing performance of non-engineered typologies in the country (“specialists”). Local experts included the municipal engineers for all six municipalities in Batanes and district engineers at the provincial level. These roles assume building regulatory responsibilities in the targeted jurisdictions encompassing extensive experience with local damage impacts from past earthquakes and typhoons in Batanes.

For the specialists, their expertise was sought because of their familiarity with the dynamics of the typologies of interest. While the impacts of hazards vary in different geographical contexts, the specialists’ judgements were considered reliable if they were acquainted with the common damage mechanisms or expected performance of these structures in the Philippine setting. We relied on academic publications and technical reports focused on seismic and wind field assessments as the basis for identifying specialists. We identified documents on Google Scholar and Scopus employing a permutation of the following keywords: “housing”, “damage”, “Philippines”, “disaster”, “shelter”, “assessment”, “wind OR typhoon”, and “seismic OR earthquake”. From this query, we shortlisted relevant documents, and then conducted literature snowballing to trace other documents not captured by the keyword search. The authors of these documents were then compiled. Finally, we shortlisted those with civil, structural, or construction engineering backgrounds.

Porter et al.^27^ developed a method to systematically solicit and aggregate experts’ opinions to derive fragility functions. This is among the methods of damage analysis developed for the ATC for its next-generation probabilistic assessment of structures adapted from the Pacific Earthquake Engineering Research Centre^27^. Unlike behavioural means of aggregating experts’ opinions wherein participants interact with each other and arrive at a consensus, this proposed method uses a mathematical approach to consolidate distinct inputs of participants^17^. Consultation with experts and combining their individual judgements provide a worthwhile basis for assessment^41^ since consensus-based approaches might reflect mere conformity among experts rather than genuine agreement^24^. While developed for seismic applications, the expert-driven method for ATC explicitly focuses on aggregating experts’ opinions. It can therefore be adaptable if the required information on component types, damage states, and engineering demand parameters (EDPs) are contextualised to the impacts of hazards of interest. Hence, we used this method in deriving both the fragility functions against the impacts of earthquakes and typhoons. The procedure asks experts to estimate the (median and lower-bound) values of a specified EDP with respect to the presented component type and damage states, alongside their self-assessed level of expertise. The use of EDP, however, is less relevant when the components of interest are low-rise non-engineered housing typologies. For example, inter-story drift ratio, being one of the most common EDPs^42,43^, is best suited for multi-story components because of the need to analyse relative translational displacement of floor levels. In this regard, it was imperative to substitute EDP with the intensity measure of a hazard.

A survey form adapted from Porter et al.^27^ was developed for this study. For earthquakes, we used the Philippine Institute of Volcanology and Seismology (PHIVOLCS) Earthquake Intensity Scale (PEIS) as the intensity measure, ranging from numeral I (weakest, “scarcely perceptible”) to X (strongest, “completely devastating”)^44^. This intensity scale, developed by PHIVOLCS, considers the common building construction types in the Philippines and the population’s perception of shaking strength, thus making it the official intensity scale in the country since 1996^45^. PEIS is comparable to the widely used Modified Mercalli Intensity (MMI) scale in that intensities I to VII represent the same effect of ground shaking, while PEIS VIII is similar to MMI VIII–IX; PEIS IX to MMI X–XI; and PEIS X to MMI XII^45,46^. We opted to use earthquake intensity instead of Peak Ground Acceleration (PGA) because the local engineers surveyed were more familiar with damage relationships with instrumental intensities. This is due to the lack of localised seismic hazard maps until recently and the limited use of PGA as a design parameter in the current National Structural Code of the Philippines. In addition, instrumental intensities can be converted to PGA when conversion equations of intensities and ground motion become available^47^ with applicability to the geographical context of interest. With the absence of such conversion equations for the Philippines for the time being, using PEIS is logical since building damageability has been long attributed to instrumental intensities within the country. Forcing the use of PGA considering such context would introduce significant, and in our view—unjustified, uncertainty in the fragility functions.

The damage states defined by FEMA for earthquake loss estimation^4,7^ were used after contextualising them to the structural properties of the identified typologies. We used these damage states because of their specificity in describing structural conditions. Thus, these well-delineated descriptions of structural failure for each damage state helped eliminate ambiguity in assessing stages of structural failure^6^. These are categorised within five damage states (DS), namely: no/very minor (DS1), minor (DS2), moderate (DS3), extensive (DS4), and complete (DS5) (see Table 1).

**Table 1:** Damage states for earthquake and typhoon impacts. Adapted from Kircher et al.^4^, FEMA^7,8^, and Vickery et al.^9^.

Damage State (DS)	Earthquake	Typhoon
DS1–No/very minor damage	None or very minor damage	None to very minor damage. Roof cover loss of less than 2% with no or limited water penetration
DS2–Minor damage	Small (≤ 1/8 inch or ≤ 3 mm) cracks or hairline cracks at corners of doors, windows, wall ceiling intersections, connections (e.g., on welds, beam and column joints, etc.), wall surfaces; spalling at a few locations (for typologies with concrete components)	Roof cover loss of 2% to 15% of the roof area but can be temporarily covered to prevent water seepage. Roof structure remains intact. Maximum of one window/door failure. No failure of wall structure but marks/dents are visible which can be repaired by painting/patching
DS3 – Moderate damage	Large (> 1/8 inch or > 3 mm) cracks at corners of doors and windows, connections (e.g., on welds, beam and column joints, etc.), wall surfaces; permanent rotation at connections are likely; spalling at wall ends (for typologies with concrete components)	Roof cover loss of above 15% to 50% of the roof area. Roof structure remains intact. Moderate window breakage. Water penetration causes some interior damage to the structure. No failure of wall structure
DS4–Extensive damage	Partial collapse, characterised by failed connections/critical elements, permanent lateral movement of floors, roof, beams, etc., extensive large/through-the-wall cracks (for concrete/masonry components) or out-of-plane failure	Roof cover loss of more than 50%. Roof structure remains intact. Major window damage. Water penetration causes extensive damage to the interior of structure. No failure of wall structure
DS5–Complete damage	Total collapse, or in imminent danger of collapse, due to failed lateral-load resisting system	Complete roof failure and/or failure of wall structure

For typhoons, we used 3-s peak gust wind speed in kilometres per hour (km/h) as the intensity measure, ranging from 0 to 400 km/h. The maximum value was capped at 400 km/h—a reasonable and realistic upper bound based on the intensity ranges of the strongest typhoons recorded in the country. For example, Typhoon Haiyan (Yolanda), one of the strongest typhoons to hit the country, had an estimated peak gust of 324 to 378 km/h^48^. The damage states for wind impacts were based on FEMA^8,9^ for the same reason that they have specific delineations of structural conditions. The damage states are categorised into five, namely: no/very minor (DS1), minor (DS2), moderate (DS3), extensive (DS4), and complete (DS5) (see Table 1).

The content of the expert survey form was divided into three parts: (1) information on the housing typologies, (2) earthquake assessment, and (3) typhoon assessment. For the first part, actual images of the typologies taken onsite were provided, alongside descriptions of the type and materials of their lateral load-resisting systems, building height, wall materials, and roof profile. For the second part, information about the recorded intensities of the 2019 earthquakes in Itbayat (and the felt intensities in surrounding municipalities)^30^ was provided to help orient the respondents about the intensity measure used. Similarly, for the third part, information about the known intensities of Typhoon Ferdie (Meranti) in 2016 and Typhoon Yolanda (Haiyan) in 2013 was supplied with a sample of the regional wind hazard map of the Philippines. For both the second and third part, the respective damage states were presented, leading to the assessment section. The experts were asked to estimate what PEIS intensity (for earthquake) and 3-s peak gust wind speed (for typhoon) will yield each of the damage states (DS1 to DS5) for each of the typologies. The experts were asked to provide both median and lower-bound intensities, following the method of Porter et al.^27,28^. We explained the median as, “What hazard intensity can bring the specified damage state to 50% of the residential structures having the same typology?” For the lower bound, it was explained as, “What hazard intensity can bring the specified damage state to 10% of the residential structures having the same typology?” Lastly, experts were also asked to rate their level of confidence with the range of estimates they provided for each typology, ranging from 1 (low) to 5 (high). Note that we used the term level of confidence instead of level of expertise to emphasise that we were interested in the trust in their estimates and not with their professional standing as this was pre-assessed before invitation using the methods described above.

The surveys were conducted online from May 2023 to July 2023. In total, we sent 57 survey invites to the roster of local experts and specialists we identified earlier. Eighteen (18) agreed to participate, five declined, and 34 did not respond. The respondents comprised seven local experts and 11 specialists. Two local experts decided to provide a single response while two specialists expressed confidence in answering only either the earthquake or typhoon assessment. We therefore collected 16 unique responses per assessment. In using expert judgement to quantify scientific uncertainty, it is suggested that 8–15 experts are reasonable, with diminishing returns becoming evident from having 20 or more participants^49^. Recent studies that used expert judgement for engineering applications have relied on this range of the number of experts involved^10,20,24^. Additionally, comprehensive insights of experts can be derived despite a limited size of cohort if and when participants are selected systematically based on their expertise aligning with the context of the assessment^49^. Finally, in the method utilised for this study, fragility functions derived through experts’ estimates are considered to be of medium quality (the highest benchmark specified) when at least three experts have ≥ 3 level of confidence^27,28^.

### Aggregation of experts’ opinion

The median and lower-bound values of experts’ estimates of hazard intensities and the corresponding levels of confidence were used as inputs for Eqs. (1, 2, 3) which are part of the method developed by Porter et al.^27,28^. These equations are based on probability encoding and expert qualification and a full transcript of their derivation is found in Porter et al.^28^.

1 $${x}_{m}= \frac{\sum_{i=1}^{N}{w}_{i}^{\mathrm{\alpha }} {x}_{mi}}{\sum_{i=1}^{N}{w}_{i}^{\mathrm{\alpha }}}$$

2 $${x}_{l}= \frac{\sum_{i=1}^{N}{w}_{i}^{\mathrm{\alpha }} {x}_{li}}{\sum_{i=1}^{N}{w}_{i}^{\mathrm{\alpha }}}$$

3 $$\beta = \frac{{\text{ln}}\left(\frac{{x}_{m}}{{x}_{l}}\right)}{1.28}$$

If Eq. (3) produces *β* < 0.4, either justify the *β*, or use Eq. 2 and Eq. 4:

4 $$\begin{aligned} \beta &= 0.4 \\ x_{m} &= {\mkern 1mu} 1.67x_{l} \\ \end{aligned}$$

where *N* = number of experts providing judgment about a value; *i* = index of experts, *i* {1, 2,…*N*}; *x*_*mi*_ = estimated median intensity measure of expert *i*;* x*_*li*_ = estimated lower-bound intensity measure of expert *i*; *w*_*i*_ = level of expertise of expert *i*; *α* = 1.5

These equations weight experts’ estimates based on their levels of confidence. The constant value of *α* renders that estimates with an assigned level of confidence of 3 are weighted five times more than just a confidence of 1, while a confidence of 5 is weighted around twice as much as 3. The constant values in Eq. (3) and (4) anchor the dispersion between the median and lower-bound values to suggest a reasonable range within these. For this study, we did not use Eq. (4) and instead just used the calculated *β*. At least for the context of this assessment, there is no plausible argument to have a definitive threshold to maintain a reasonable gap between the solicited median and lower-bound values. For example, a difference of just 1 PEIS intensity can have pronounced implications for housing damage, as with a 0.5 difference, depending on how these structures are built. For wind speeds, a difference of 10 km/h might already bring lower-bound and median probabilities of damage to certain typologies, while for others, higher wind speeds bring more pronounced housing damage.

### Plotting of fragility functions

Fragility functions define the probability of a damage state *ds* being exceeded or reached for a component, given a particular value of intensity measure *im*, such that P[DS ≥ ds | IM = im]. In this expression, lowercase notations indicate particular values of the uppercase variables. Fragility functions are most commonly idealised through a lognormal cumulative distribution function^5,6,50^ (see Eq. 5). Each fragility function needs a median value (*x*_*m*_) of the intensity measure representing the threshold and the variability of a damage state, and a logarithmic standard deviation (*β*) describing the total variability of the damage states^4^.

5 $$P\left[DS\ge ds \right|IM=im] = \phi \left(\frac{{\text{ln}}(im/{x}_{m})}{\beta }\right)$$

where *ds* = specific damage state; *im* = particular value of intensity measure; *ϕ* = standard normal cumulative distribution function; *x*_*m*_ = median value of distribution (as derived in Eq. 1); *β* = logarithmic standard deviation (as derived in Eq. 3).

In plotting the fragility functions, we used the *plnorm()* function in RStudio which idealises the lognormal cumulative distribution function. We used the calculated *x*_*m*_ and *β* values from Eqs. (1) and (3). For the *x*_*m*_ inputs, we first calculated their lognormal values to standardise on the log scale before feeding them into the function. For one typology (LW-B), we encountered a minor crossing of two functions (damage states). Crossing of curves implies a meaningless negative probability and this was addressed using Eqs. (6) and (7) to adjust *x*_*m*_ and *β* values as proposed by Porter et al.^27,28^. Where functions *i* and *j* cross having medians *x*_*mj*_ > *x*_*mi*_ and *β*_*i*_ ≠ *β*_*j*_, the adjusted values *x’*_*mi*_ and *β’*_*i*_ were calculated.

6 $${\beta^{\prime}}_{i}= \frac{1}{N} \sum_{i=1}^{N}{\beta }_{i}\, for\, all\, i$$

7 $${x\mathrm{^{\prime}}}_{mi}={\text{exp}}(1.28(\mathrm{\beta^{\prime}}- {\beta }_{i})+\mathrm{ln }{x}_{mi})$$

### Ethics and inclusion statement

All procedures performed involving human subjects were in accordance with the Human Research Ethics Committee of The University of Sydney under the approved protocol 2022/705. The field study site was located on the ancestral domain of the Indigenous people of Itbayat. A free prior-informed consent was obtained from the National Commission on Indigenous Peoples under Certificate of Precondition R2-IKSP-2022–12-21.

## Results

### Identified housing typologies

#### Unreinforced masonry

**Figure 2:**
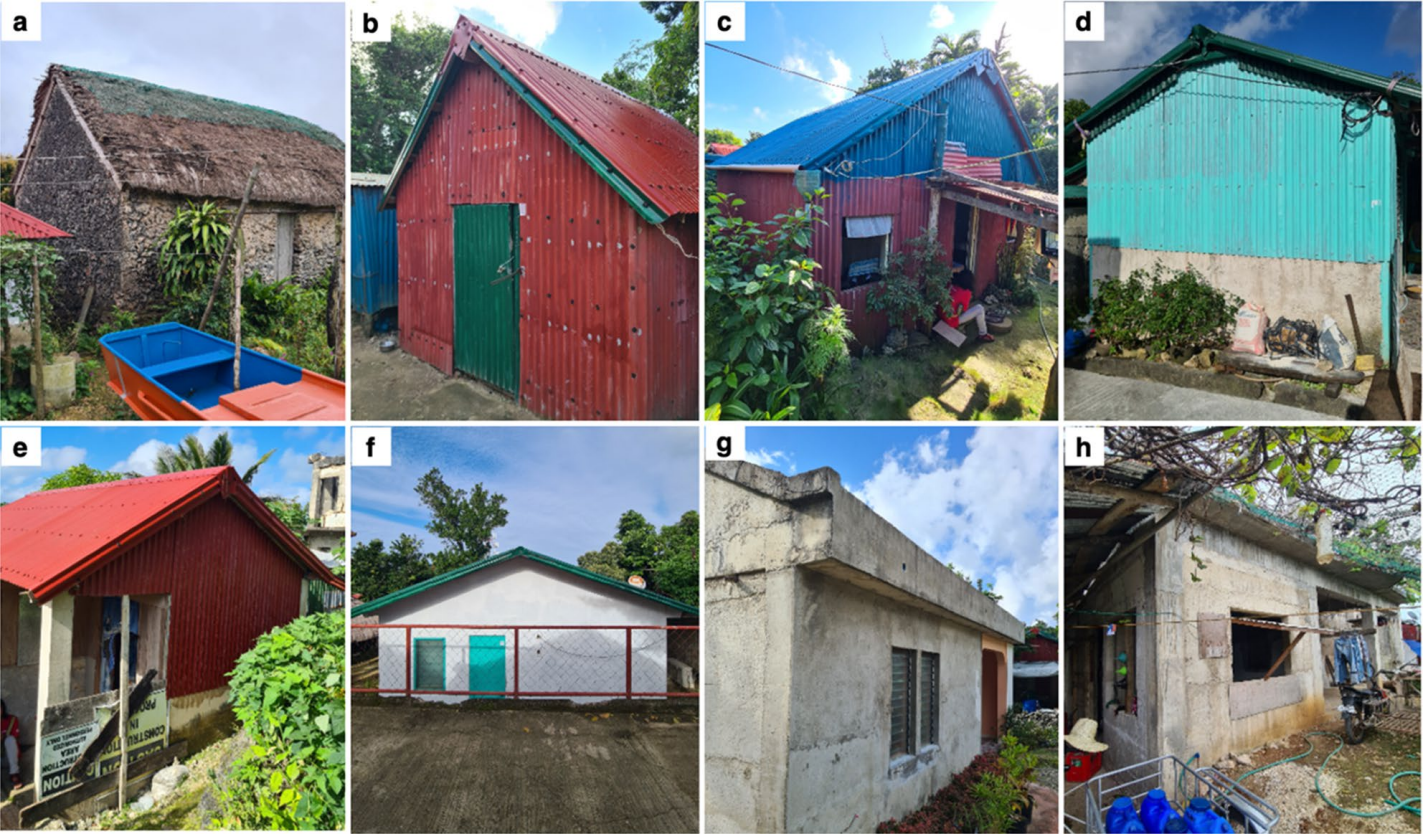
The surveyed housing typologies in Itbayat. (**a**) Unreinforced masonry (URM). (**b**) Lightweight with wooden posts (LW-A). (**c**) Lightweight with steel posts (LW-B). (**d**) Semi-concrete with steel posts (SC-A). (**e**) Semi-concrete with RC posts (SC-B). (**f**) RC structure with lightweight roofing (RC-A) where in some instances, inside gutters are common to conceal the edges of roofing sheets as shown in (**g**). (**h**) RC structure with RC slab roofing (RC-B).

**Figure 3:**
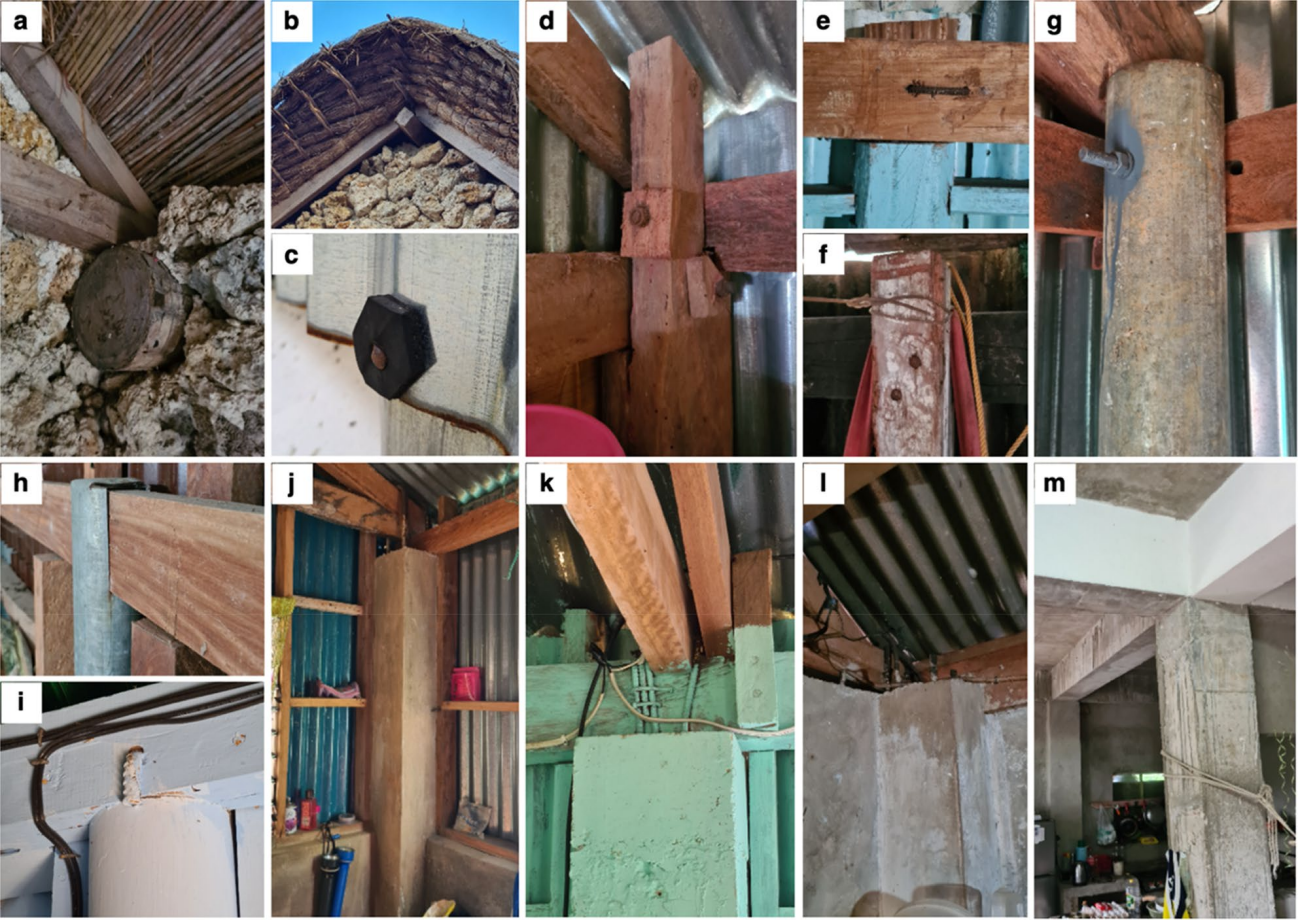
Structural details documented across the typologies surveyed. (**a**) Roof-to-wall connection where thick logs are embedded in stone walls and (**b**) layered thatch (cogon) roofing with reed matting are features of URM. (**c**) Improvised rubber washers for nail fasteners are used for typologies with CGI sheets. For LW-A, common post-and-beam connections are (**d**) bolted, (**e**) hooked with reinforcing steel bars, and (**f**) nailed. For LW-B and SC-A, common post-and-beam connections are (**g**) bolted, (**h**) notched and clipped, and (**i**) hooked with reinforcing steel bars. For SC-B, typical post-and-beam connections are (**j**, **k**) dowels wrapped around beams and roof members. For RC-A, (**l**) roof-to-wall connections are common to be via dowels from reinforcing steel bars wrapping the wooden roof members. For RC-B, (**m**) beams and columns are reinforced concrete supporting the slab roof.

Before the series of earthquakes in 2019, unreinforced masonry (“URM”) dwellings were the vernacular housing archetype in the municipality of Itbayat like elsewhere in the province of Batanes. This typology is characterised by thick (0.80—1 m in width) coral limestone walls bounded by quicklime mortar and roofed with layered cogon. Unique in Batanes, this typology is of Spanish colonial influence adapted by the Ivatans in response to frequent typhoons^38,40^. With wind-induced damages common in this context, features to safeguard the structure include solid wooden panels with shutters to cover openings, a low stature of the structure for better wind resistance, and a blank wall facing the direction where wind blows strongest. Whilst responsive against wind impacts as per locals’ experience, this typology proved vulnerable to ground shaking—evident on the extensive damage experienced following the maximum intensity of PEIS VII from the 2019 earthquakes^30^. A conservative estimate based on field data suggests that there are fewer than ten structures remaining of such typology in Itbayat, though much can still be seen in most municipalities in Batanes. Post-earthquake, the household-led reconstruction resulted in a departure from the vernacular construction styles. New typologies emerged due to regulatory restrictions affecting traditional resource extraction of building materials (e.g., hardwood, limestones, and cogon), the favourability of contemporary construction methods, and the presence of modern shelter materials donated by organisations. Six prominent housing typologies were identified replacing URM which account for 91% of the surveyed building stock. These include variations of lightweight, semi-concrete, and reinforced concrete (RC) dwellings (see Figs. 2 and 3).

#### Lightweight

Two variations of lightweight structures were identified, both having post-and-beam lateral load-resisting systems. The first variation (“LW-A”) features timber beams and timber columns for primary (corner) posts, where the columns do not have footings and are only driven underground. The beam-to-column connections vary with the use of bolts, nails, and improvised hooks made from reinforcing steel bars (RSB). The second variation (“LW-B”) of lightweight structures has primary (corner) posts made of 4-inch to 5-inch diameter steel pipes with RC footing and with timber beams. These components are connected via bolts, improvised hooks made from RSB, and in some instances, the beams are notched and clipped inside the steel pipes. Both lightweight typologies are one-story structures having gable roof profiles, where their roof eaves rarely exceed 300 mm. Roof and walls are enveloped by corrugated galvanised iron (CGI) sheets fastened every other one or two corrugations on wooden frames using common wire nails with improvised rubber washers. Additional timber posts as intermediate supports are used. CGI or plywood covers for door openings are common and the same for windows alongside jalousie (louvred) glass. Of the surveyed residential building stock, 17% and 25% account for LW-A and LW-B, respectively.

#### Semi-concrete

For semi-concrete structures, two variations were surveyed, both having hybrid lateral load-resisting systems. These typologies have post-and-beam systems in combination with half-height RC walls at the base of the structure providing additional lateral stiffness. The first variation of semi-concrete structure (“SC-A”) has primary (corner) posts made of 4-inch to 5-inch diameter steel pipes with RC footing and with timber beams. The beam-to-column connections vary with the use of bolts, improvised hooks made from RSB, and in some instances, the beams are just notched and clipped inside the steel pipes. The second variation (“SC-B”) has RC columns with RC footing. The columns have dowels on top used to wrap and fasten the timber beams (and sometimes together with roof members). Both semi-concrete typologies are one-story structures having gable roof profiles, where their roof eaves rarely exceed 300 mm. Roof and walls are enveloped by CGI sheets fastened every other one or two corrugations on wooden frames using common wire nails with improvised rubber washers. Additional timber posts as intermediate supports are used. CGI or plywood covers for door openings are common and the same for windows alongside jalousie (louvred) glass. SC-A has a 17% share of the surveyed building stock, while SC-B has 14%.

#### Reinforced concrete

Two types of RC structures were identified—one with lightweight roofing (“RC-A”) and the other one with RC slab roofing (“RC-B”). RC-A has a gable roof configuration with eaves rarely exceeding 300 mm. The CGI roof panels are fastened to wooden roof members using common wire nails with improvised rubber washers. Meanwhile, RC-B has a flat slab with eaves typically exceeding 300 mm. Both RC typologies are one-story structures having RC posts (including footings), beams, and walls. As such, these are considered to have hybrid lateral load-resisting systems characterised by their moment frame connections in combination with RC walls providing additional lateral stiffness. CGI or plywood covers for door openings are common and the same for windows alongside jalousie (louvred) glass. Of the surveyed residential building stock, 9% are RC-A and 9% are RCB.

All the typologies identified in this study are considered non-engineered due to limited regulatory building oversight. For URM, building codes and standards were not yet in place, or perhaps limited, when these structures were constructed (circa ~ 1900s). In 2019 after the earthquakes, the housing reconstruction phase coincided with the typhoon season, and although the need for a building permit was not explicitly waived, the sense of urgency dictated the thrust of the household-led rebuilding influenced by the resources available to households. For example, those who chose to build LW-A and LW-B initially envisioned these as temporary, intending to build more permanent dwellings later, but financial limitations precluded this from happening. Nevertheless, building materials were carefully selected in some instances. For example, local hardwood was favoured over commercial timber for main structural elements such as rafters and beams. For those who chose to build concrete typologies, imported commercial aggregates from mainland Luzon were desired for their quality. However, since the importation of materials would inflate building costs, households used local aggregates (crushed coral limestones) for walls while commercial aggregates were used for beams and columns.

### Fragility functions

**Figure 4:**
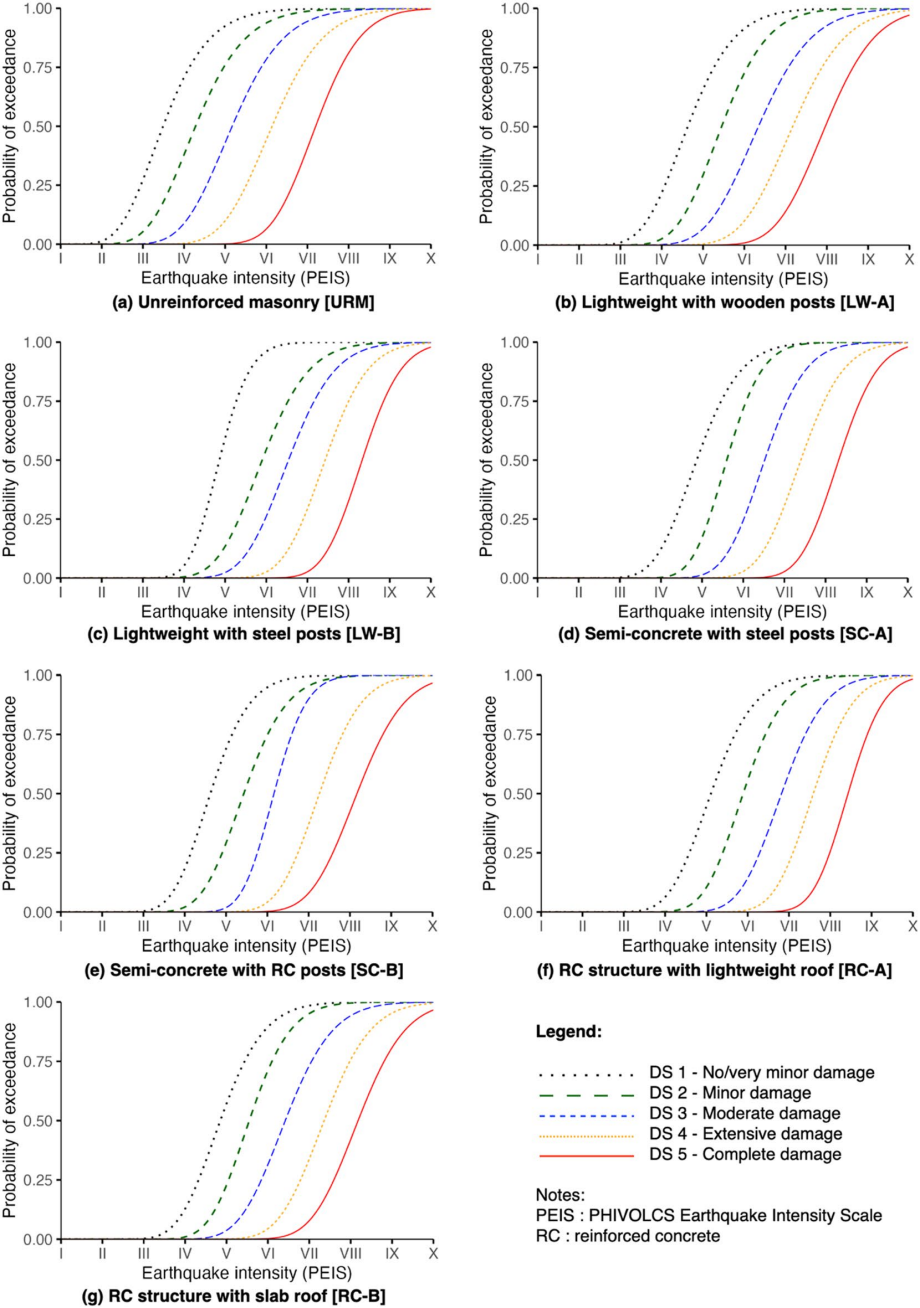
Fragility functions against seismic impacts derived from experts' estimates.

**Figure 5:**
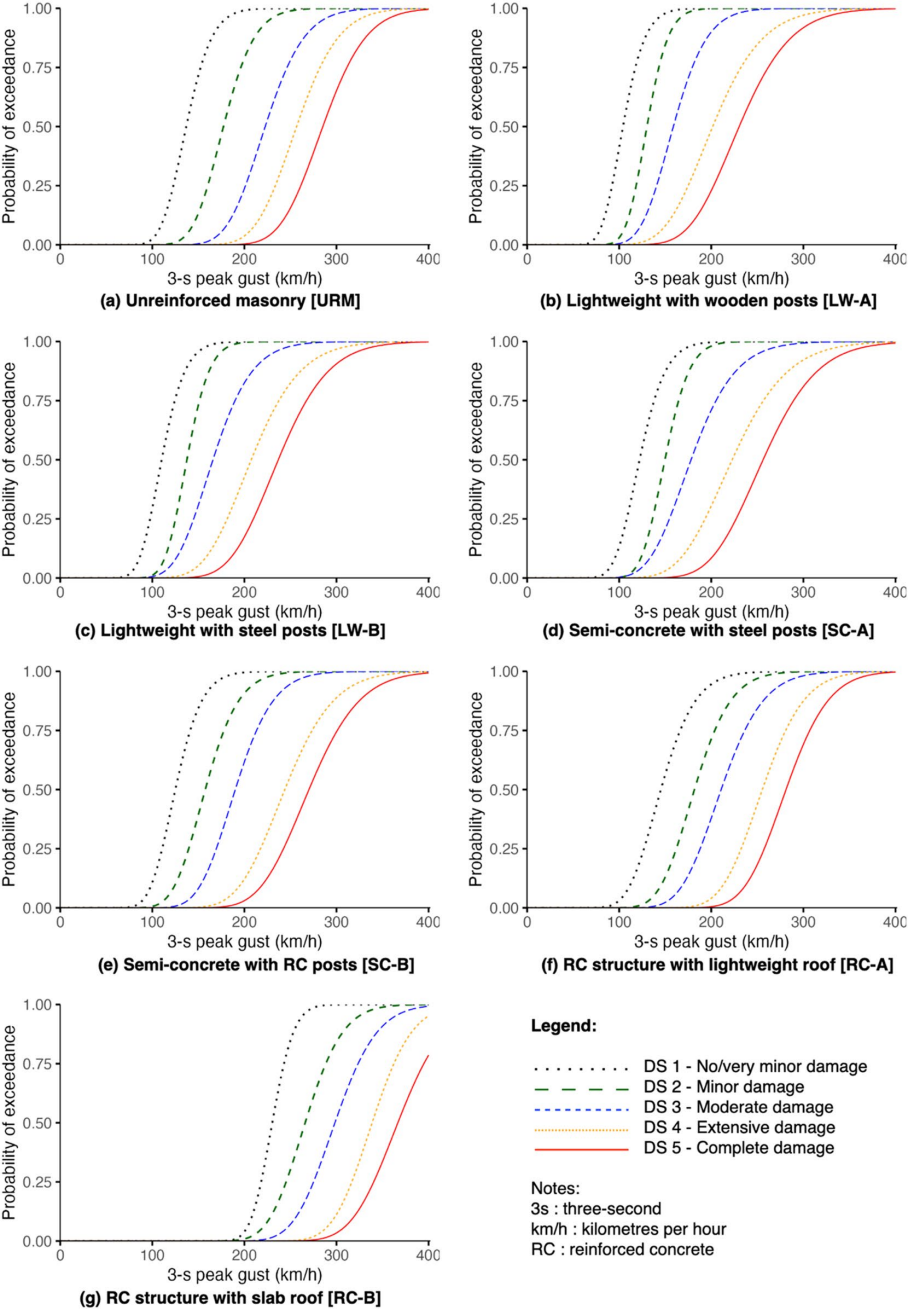
Fragility functions against wind impacts derived through experts' estimates.

The fragility functions derived in this study, presented in Figs. 4 and 5, are based on the calculated *x*_*m*_ and *β* values shown in Table 2. For seismic impacts, URM has a 27% probability of reaching or exceeding very minor damage (DS1) at PEIS III. This intensity is of weak shaking comparable to the vibration of a passing light truck. The rest of the typologies have negligible probabilities (almost 0%) to any damage state at this intensity. At PEIS V, described as strong shaking with a rocking effect on buildings, all typologies except for URM show a 45% to 69% probability of meeting or exceeding DS1 and a likelihood of ≤ 30% of DS2 (minor damage) and DS3 (moderate damage). Meanwhile, at a very destructive shaking (PEIS VIII) where many well-built buildings are expected to be considerably damaged, URM structures have a 95% probability of reaching or exceeding DS4 (extensive damage) and 81% for complete damage (DS5). For lightweight typologies, there are 78% (LW-A) and 75% (LW-B) probabilities of meeting or exceeding DS4, and 52% (LW-A) and 33% (LW-B) likelihood for DS5. For semi-concrete typologies, still considering PEIS VIII, there is 76% (SC-A) and 81% (SC-B) probabilities of reaching or exceeding DS4, and 33% (SC-A) and 42% (SC-B) probabilities for DS5. For RC typologies, DS4 has 70% (RC-A) and 76% (RC-B) chances of being reached or exceeded, while 25% (RC-A) and 42% (RC-B) for DS5. Generally, URM tends to be the most vulnerable typology to seismic impacts while the other typologies perform better, either because of low structure weights (LW-A, LW-B), more robust lateral force resisting systems (RC-A, RC-B), or a combination of these features (SC-A, SC-B).

**Table 2:** *x*_*m*_ and *β* values of the fragility functions.

Typology	Earthquake					Typhoon				
	DS1	DS2	DS3	DS4	DS5	DS1	DS2	DS3	DS4	DS5
URM	*x*_*m*_*:* 3.49	4.28	5.17	6.14	7.20	*x*_*m*_*:*136.87	177.26	222.60	256.70	285.18
	*β:* 0.25	0.22	0.19	0.16	0.12	*β:* 0.16	0.15	0.15	0.14	0.13
LW-A	*x*_*m*_*:* 4.63	5.45	6.34	7.17	7.96	*x*_*m*_*:*104.12	130.13	158.80	201.53	230.30
	*β:* 0.19	0.16	0.16	0.14	0.12	*β:* 0.18	0.14	0.18	0.21	0.19
LW-B	*x*_*m*_*:* 4.86	5.90	6.54	7.43	8.33	*x*_*m*_*:*110.18	137.59	165.86	207.47	236.83
	*β:* 0.12	0.15	0.14	0.11	0.09	*β:* 0.18	0.14	0.20	0.20	0.18
SC-A	*x*_*m*_*:* 4.87	5.60	6.52	7.40	8.32	*x*_*m*_*:*122.14	149.67	178.05	222.68	256.26
	*β:* 0.17	0.12	0.12	0.11	0.09	*β:* 0.18	0.14	0.21	0.21	0.18
SC-B	*x*_*m*_*:* 4.62	5.43	6.16	7.26	8.17	*x*_*m*_*:*125.02	157.52	190.12	243.92	270.10
	*β:* 0.16	0.15	0.10	0.11	0.11	*β:* 0.18	0.18	0.17	0.17	0.16
RC-A	*x*_*m*_*:* 5.11	5.89	6.82	7.59	8.43	*x*_*m*_*:*145.23	181.70	210.73	255.46	281.16
	*β:* 0.16	0.13	0.12	0.10	0.08	*β:* 0.20	0.17	0.17	0.14	0.13
RC-B	*x*_*m*_*:* 4.85	5.55	6.42	7.36	8.18	*x*_*m*_*:*230.27	266.00	298.31	338.73	366.65
	*β:* 0.17	0.14	0.14	0.12	0.11	*β:* 0.08	0.12	0.12	0.10	0.11

While URM might be vulnerable to seismic impacts, it generally tends to perform better against wind alongside RC typologies. At 100 km/h 3-s peak gust, URM has a 2% likelihood of reaching or exceeding DS1, RC-A has a 3% probability and RC-B has a negligible probability. At the same wind intensity, lightweight typologies show 41% (LW-A) and 30% (LW-B) probabilities of meeting or exceeding DS1, while semi-concrete typologies show 13% (SC-A) and 11% (SC-B) probabilities of exceedance. Considering a 200 km/h peak gust, URM, RC typologies, and SC-B have very low to negligible probabilities of reaching or exceeding DS5 unlike LW-A, LW-B, and SC-A where there are 22%, 17%, and 8% chances of meeting or exceeding DS5, respectively. This wind intensity corresponds roughly to an estimated 20-year return period in the Province of Batanes, meaning that it has a 5% possibility to happen in a given year, based on the regional severe wind hazard maps for the Philippines^51^. At 300 km/h, corresponding to a 500-year return period (0.2% chance to happen in a given year), URM structures have 87% and 65% probabilities of reaching or exceeding DS4 and DS5, respectively. RC-A is expected to perform similarly with 87% and 69% probabilities of DS4 and DS5, respectively. Meanwhile, RC-B shows an 11% probability of reaching or exceeding DS4 and a 3% probability for DS5. For lightweight typologies, DS4 has a likelihood of 97% (LW-A and LW-B), while DS5 corresponds with 92% (LW-A) and 91% (LW-B). For semi-concrete typologies, DS4 shows 92% (SC-A) and 89% (SC-B) probabilities of exceedance, and 81% (SC-A) to 74% (SC-B) for DS5. At 400 km/h, all typologies have > 99% probability of exceedance for DS5, except for RC-B having a 79% likelihood of reaching or exceeding this damage state.

Studies on the derivation of fragility functions in the Philippines are presently very limited. Cross-comparison of our functions to the available studies is challenging given the differences of how typologies and damage states are characterised including the nuanced assumptions considered in deriving these functions. Given these limitations, we were only able to draw comparisons to the works of Pacheco et al.^52^ and Naguit et al.^53^ on the fragility functions derived for lightweight, wooden-framed structures. This typology is comparable to LW-A (lightweight with wooden posts) in this study characterised by wooden post and beam systems. Other typologies from these studies exhibit different structural characteristics from the ones we surveyed. For example, low-rise concrete-framed typologies described by both studies cannot be used for direct comparison since in their works, these are described as concrete moment-framed structures with (hollow block) masonry walls whereas those surveyed in this study were concrete moment-framed structures with RC walls, adding lateral stiffness to the structure. Pacheco et al.^52^ derived their functions using a hybrid approach (computational and heuristic) while Naguit et al.^53^ generated their functions using empirical methods using data collected by communities.

**Figure 6:**
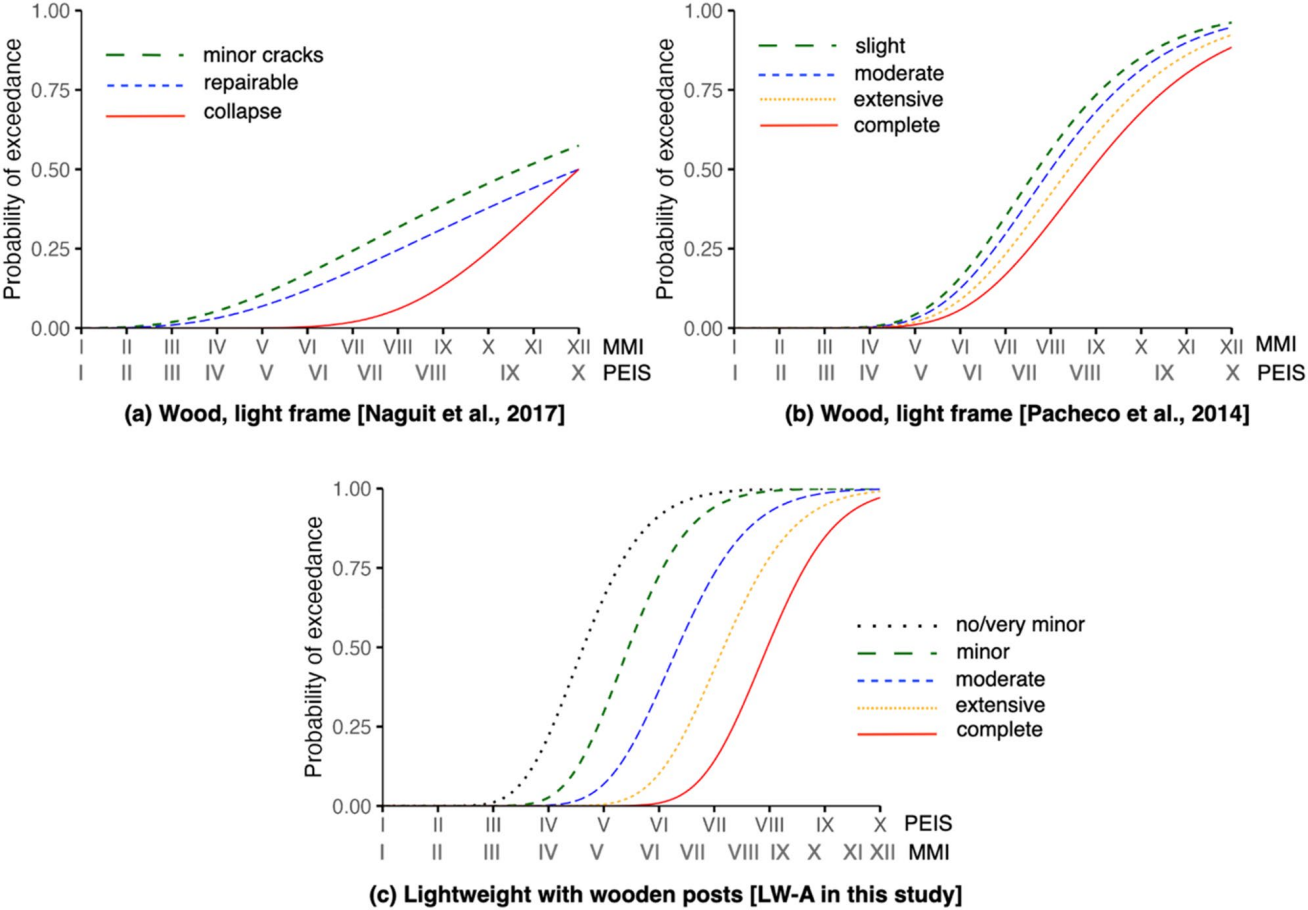
Comparison of seismic fragility functions to other studies for lightweight, wooden-framed typology. (Note: PEIS stands for PHIVOLCS Earthquake Intensity Scale while MMI stands for Modified Mercalli Intensity).

**Figure 7:**
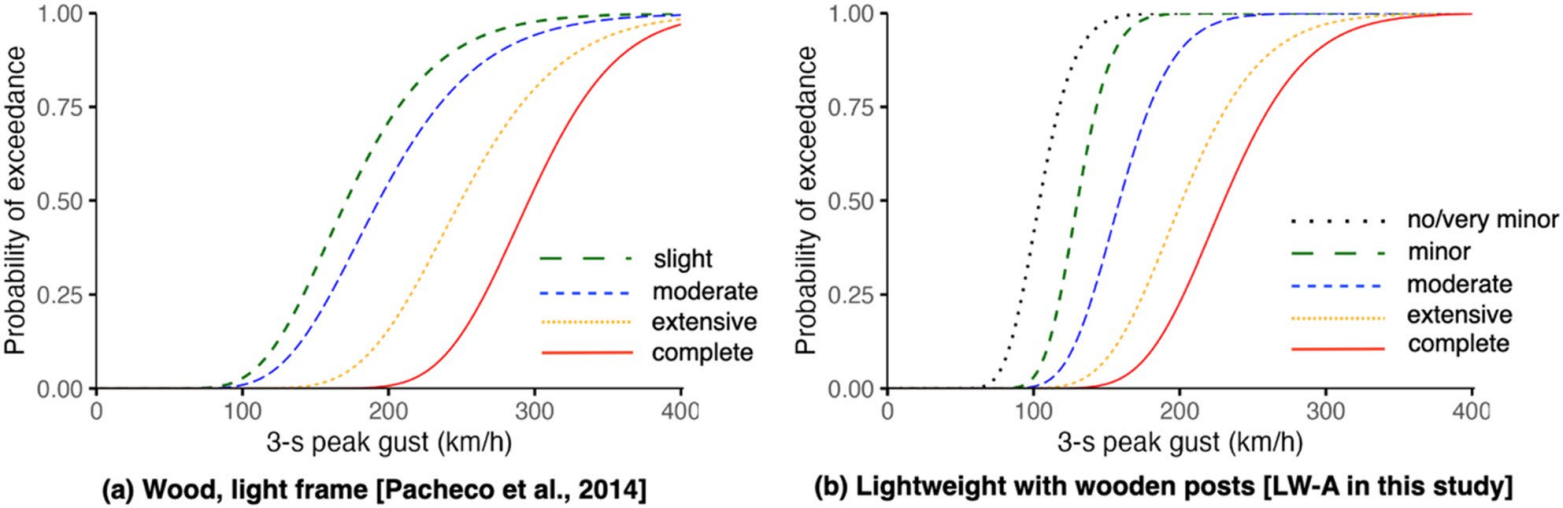
Comparison of wind fragility functions to other studies for lightweight, wooden-framed typology.

Comparing seismic fragilities of lightweight wooden structures (see Fig. 6), our functions provide higher probabilities for complete damage or collapse. Whereas Naguit et al.’s^53^ functions estimate ~ 50% probabilities of exceedance for collapse and Pacheco et al.’s^52^ functions estimate ~ 85% for complete damage at the maximum seismic intensity possible (PEIS X / MMI XII), our functions assume complete damage at this intensity close to 100% probability of exceedance. Our functions also indicate that slight damage may start to manifest between Intensities II and III, close to Naguit et al.’s^53^ which suggest minor cracks starting to manifest around Intensity II. Meanwhile, Pacheco et al.’s^52^ functions indicate slight damage starting to become possible at Intensity IV. For wind comparisons (see Fig. 7), both our functions and Pacheco et al.’s^52^ suggest > 75% probabilities of exceedance for complete damage for wind speeds greater than 350 km/h. For both functions, slight/minor and moderate damage start to arise around 100 km/h but ours have steeper curves suggesting a more rapid change in probabilities with the increase of wind speed. In comparing functions, caution should be taken since differences can emanate from the varied characterisations adopted by authors. For example, the use three-tier damage scale (e.g., Naguit et al.^53^) can result to a different set of functions when applying more granular observations to visualise or represent five damage states.

Benchmarking the accuracy of the functions derived in this study is difficult as these are the first to be derived for this geographical context. In the interim, these functions can be considered as medium quality having satisfied the criteria of Porter et al.^27,28^ to have at least three experts whose confidence ratings are ≥ 3. For the expert-driven approach in ATC-58, only low and medium-quality benchmarks are provided in reporting on the quality of the derived functions. At least 68% of the responses for every typology for each hazard received confidence ratings of 3 or greater (out of 5). As shown in Table 3, there was variation in experts’ confidence in their assessments. The median of confidence level for wind assessments for each typology is 3 across all typologies. Meanwhile, for seismic assessments, heavier typologies (URM, RC-A, and RC-B) have a median confidence rating of 4 while the rest, which are the lighter weight counterparts, have a median confidence level of 3. Considering experts’ unweighted (or raw) inputs, standard deviations across damage states for earthquake intensities range from PEIS 0.83–PEIS 1.54, while for typhoon wind speeds, they range from 43.29 to 105.65 km/h. The minimum and maximum values of the consolidated estimates from both local experts and specialists are presented in Table 3 and these values show the variation of inputs solicited for this assessment.

**Table 3:** Ranges of consolidated experts' estimates shown through minimum and maximum values, including the median.

			Earthquake intensity (PEIS)							Typhoon intensity (km/h)						
			URM	LW-A	LW-B	SC-A	SC-B	RC-A	RC-B	URM	LW-A	LW-B	SC-A	SC-B	RC-A	RC-B
Confidence levels*		Min	1	1	3	1	1	1	1	1	1	1	1	1	1	1
		Max	4	4	4	5	5	5	4	5	5	5	5	5	5	5
		Median	4	3	3	3	3	4	4	3	3	3	3	3	3	3
DS 1	Median IM**	Min	2	1	3	2	2	4	3	50	30	30	60	60	45	70
		Max	6	4	8	8	7	7	7	300	225	225	300	300	300	400
		Median	3	3	5	4	4	5	4.5	100	100	100	100	100	120	150
	Lower bound IM	Min	1	1	2	1	1	1	2	30	20	25	50	55	30	60
		Max	5	7	8	7	6	6	5	250	200	200	250	250	250	400
		Median	2	3	4	3	3	4	4	80	60	70	80	80	100	130
DS 2	Median IM	Min	3	3.5	4	3	2.5	4.5	3	70	40	40	75	80	55	90
		Max	6	8	8	8	8	8	8	325	250	250	325	325	325	400
		Median	4	5	6	5	5	5.75	5.5	150	120	126	135	140	150	200
	Lower bound IM	Min	2	2	3	2	1	2	2	40	25	30	60	70	45	70
		Max	5	7	8	8	7	7	6	275	225	225	275	275	275	400
		Median	3	4	5	4	4	5	5	120	100	100	100	100	125	155
DS 3	Median IM	Min	3	4	4.5	3.5	3	5	4	75	60	60	90	90	75	160
		Max	7	9	9	9	8	9	8	350	275	275	350	350	350	400
		Median	5	6	6.75	6	6	7	6.75	180	155	155	160	180	200	300
	Lower bound IM	Min	2	3	4	3	2	4	2	50	40	45	60	60	50	100
		Max	6	8	8	8	8	8	7	300	250	250	300	300	300	400
		Median	4	5	5.5	5	5	6	5.5	160	115	120	125	145	150	200
DS 4	Median IM	Min	4	5	6	4	4	6	5	80	90	90	140	140	90	220
		Max	8	10	10	9	9	10	9	375	300	300	375	375	375	400
		Median	6	7	7.25	7	7	7.5	7.5	200	200	200	200	205	240	350
	Lower bound IM	Min	3	3	4	3	2	4	3	70	80	90	100	100	75	160
		Max	7	9	9	8	9	9	8	325	275	275	325	325	325	400
		Median	5	6	6.25	6	6	7	6.5	175	150	150	150	175	200	300
DS 5	Median IM	Min	5	6	6.5	5	5	7	6	90	110	110	150	150	100	250
		Max	9	10	10	10	10	10	10	400	325	325	400	400	400	400
		Median	7	8	8	8	8	8	8	250	245	250	250	240	260	400
	Lower bound IM	Min	4	5	6	4	3	5	5	60	90	100	100	100	85	180
		Max	8	9	9	9	9	10	9	375	300	300	350	350	350	400
		Median	6.5	7	7.25	7	7	7.5	7.25	200	180	185	200	200	222.5	325

*Confidence levels are ordinal ratings from 1 to 5.

**IM stands for intensity measure. For earthquake, it is expressed in PHIVOLCS Earthquake Intensity Scale (PEIS). For typhoon, it is expressed in kilometres per hour (km/h).

To summarise, respondents were more confident in their assessments of heavier typologies for both hazards. However, for typhoon hazards, variance of responses increased from light to heavy typologies, suggesting wider variation in expert assessments. In general, we argue that the variability of experts’ inputs should be considered an advantage for the context of this assessment because such variability encompasses the broad scenario-based observations of housing performance. This consideration is beneficial most especially where construction resources are used less prescriptively against building codes and standards requiring less straightforward appraisal of housing performance.

## Discussion

In conducting the expert-driven approach of deriving fragility functions, we gleaned three insights to consider when using this heuristic alternative for damage analysis. These insights include how disaster sub-culture influences experts’ estimates, some caveats to be aware of when relying on hazard intensities published by agencies, and the logistical challenges of soliciting experts’ opinions.

In areas like Batanes where people have perennial encounters with hazards, disaster preparedness practices instinctively emerge to become part of their disaster sub-culture. For example, before a typhoon hits, houses are tied up with ropes to secure their envelopes (a practice called *kap’yakuyakut* in Itbayat and *kapanpet* in mainland Batanes). Meanwhile, windows are covered with an additional layer of improvised shutters (*tapangko*). During the solicitation of experts’ opinions, a local expert commented that his estimates were guided by how the houses perform in conjunction with these prevailing disaster preparedness practices in the area. These considerations provide a layered understanding of housing performance embedded in local settings—and why the development of localised fragility functions is important. Conventional approaches to damage analysis would suggest equal, unifying assumptions for assessments. However, understanding these local practices requires context and nuance not captured by decoupling building elements and preparedness activities. Commanding local experts to “disregard” such considerations is counterintuitive given that the core basis of their estimates is on actual observations. Beyond the expert-driven approach, there is an opportunity to consider these types of non-traditional measures in computational fragility modelling done experimentally. These disaster preparedness practices will also have an impact on future empirical data collected in Batanes since housing damage will likely be reflective of the housing performance in conjunction with such practices.

Damage estimates based on experts’ opinions are guided by actual observations of housing performance based on past disaster events. These observations are most likely referenced with impactful hazard intensities as broadcasted by agencies. Since damage estimates are relative or “framed” within agency-interpreted intensities, any errors, limitations, or discrepancies in how these intensities are interpreted affect the experts’ estimates. On the survey forms, we provided information sheets that contained ranges of hazard intensities of notable disaster events in the Philippines from different data sources to guide respondents in situating their estimates. The intention was to confine their estimates within reasonable hazard intensities that were actually recorded. However, in some instances, there is no definite historical upper bound of hazard intensities for wind events due to previous data limitations. For example, recorded peak gusts of Typhoon Meranti (Ferdie) in 2016 are unavailable in Itbayat where it made landfall^54^. Likewise, peak gusts of Typhoon Haiyan (Yolanda) were not recorded across some weather stations in the Visayas region due to the damage sustained by the weather instruments^48,55,56^. To compensate for such limitations, intensity readings of neighbouring international weather agencies were used. Additionally, estimated intensities evident from impact signs to infrastructure as derived in some studies were referenced^55–57^. However, without officially recorded intensity readings of some of the largest typhoons in the Philippines, estimates will always be confined within the proxied or assumed values. For seismic impacts, a factor to consider as raised by one specialist is the possibility of circular logic between PEIS intensities and damage states. That is, PEIS ground shaking is at least partly pegged to certain descriptors of building damage.

On the use of the expert-driven approach as a viable alternative where analytical and empirical data are currently impractical to acquire, we realise that expertise can also be a rare commodity. Local expertise might be limited at a municipal level especially in small, geographically remote areas. We have addressed this issue by broadening the jurisdiction to the provincial level where district engineers compensated for this limitation. Additionally, while there is a reasonable number of specialists who could be prospective respondents, their availability was not guaranteed, and response rates mainly dictated the sampling of the respondents. Therefore, robustly qualifying who gets involved was the priority more than reaching a certain quota of respondents with a focus on quality over quantity of participant selection.

These three insights guided our derivation of the first-generation fragility functions in Batanes. The non-engineered nature of the typologies surveyed makes it a challenge to definitively assess the variability inherent to this building stock. This study, however, advanced this pursuit by acknowledging the nuanced characteristics of non-engineered typologies, which could have otherwise been categorised within existing generic taxonomies. In the Philippines, previous efforts to generate fragility or vulnerability functions for residential typologies have been conducted for Greater Metro Manila Area^52^, Bohol^53^, and Cebu^58^, with contributions in the GEM database. This study branched out further north to consider the rural context of a small island geography which has been pressured to address multi-hazard housing safety due to the competing impacts of extreme events. The functions derived are fundamental tools for practitioners to understand the vulnerability of non-engineered structures and offer targeted structural mitigation and preparedness programmes contextualised in the area. It is suggested that when more empirical data becomes available, the possibility of combining new and existing functions should be explored^50,59^.

## Conclusion

Using fragility functions in risk assessments is fundamental to model anticipated structural housing damage against the impacts of natural hazards. We used an expert-driven approach to derive wind and seismic fragility functions for the Province of Batanes. Theoretically, this study provides new estimates of the performance of non-engineered housing in the Philippines, which would traditionally be discounted by conventional fragility assessments relying on generic building taxonomies. This region-specific evaluation is a step to advance more localised risk assessments. Practically, the set of fragility functions derived in this study is seen to assist practitioners in exploring risk preparedness and mitigation measures applicable in Batanes. On a national scale, we fill gaps on the lacking representation of building archetypes in rural and remote areas as most attention to understanding building-level risk reduction has, to date, focused in urban centres where data are more accessible. Our functions lay a foundation for more holistic and contextualised approaches to risk modelling nationwide considering the inclusion of neglected geographic areas. As new damage data and analysis emerge in the future, our functions can aid hybrid approaches to generate the next generation of fragility estimates.

## Data Availability

The datasets used and analysed in this study are available from the corresponding author upon reasonable request.
